# Correction: Radical ring-opening polymerization of sustainably-derived thionoisochromanone

**DOI:** 10.1039/d3sc90098c

**Published:** 2023-06-12

**Authors:** Emily A. Prebihalo, Anna M. Luke, Yernaidu Reddi, Christopher J. LaSalle, Vijay M. Shah, Christopher J. Cramer, Theresa M. Reineke

**Affiliations:** a Department of Chemistry, University of Minnesota 207 Pleasant St. SE Minneapolis MN 55455 USA treineke@umn.edu; b Underwriters Laboratories Inc. 333 Pfingsten Rd. Northbrook Illinois 60620 USA

## Abstract

Correction for ‘Radical ring-opening polymerization of sustainably-derived thionoisochromanone’ by Emily A. Prebihalo *et al.*, *Chem. Sci.*, 2023, https://doi.org/10.1039/d2sc06040j.

The authors regret that incorrect details were given for ref. 28 in the original article. The correct version of ref. 28 is given below as ref. 1.

The Royal Society of Chemistry apologises for these errors and any consequent inconvenience to authors and readers.

## Supplementary Material
